# Supplementary material for: Adrenal tropism of SARS-CoV-2 and adrenal findings in a post-mortem case series of patients with severe fatal COVID-19
Source: Nat Commun. 2022 Mar 24;13:1589. doi: 10.1038/s41467-022-29145-3 (PMC8948269; doi:10.1038/s41467-022-29145-3)
Supplement: Supplementary file 1 — Supplementary Information [file 41467_2022_29145_MOESM1_ESM.pdf]

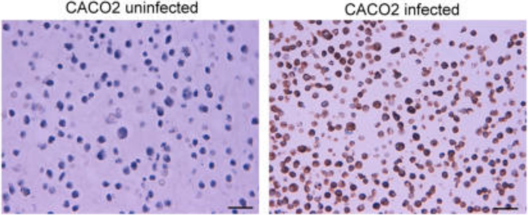

Supplementary Figure 1. Immunohistochemistry of s-SARS-CoV-2 of cell blocks of CACO2 uninfected and infected with SARS-CoV2 (scale bar = 10 μm).

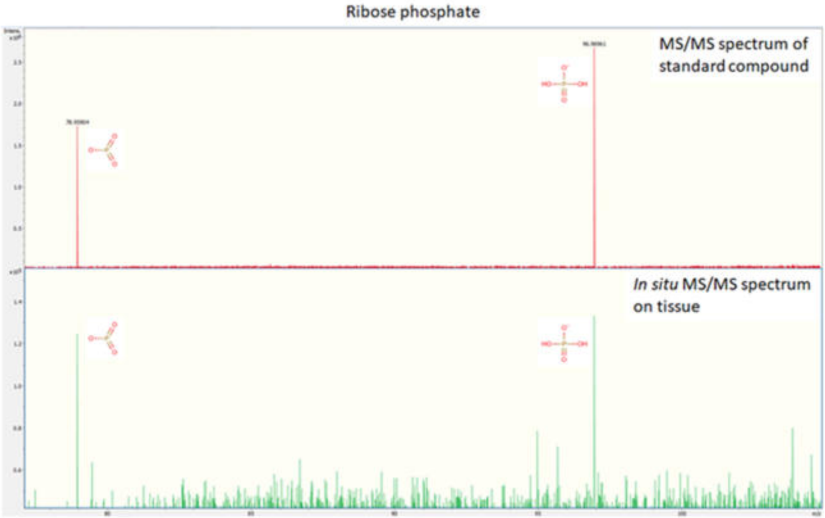

Supplementary Figure 2. Ribose phoshate was identified by in-situ MS/MS experiments comparing the observed MS/MS spectra (green) with standard compounds (red).

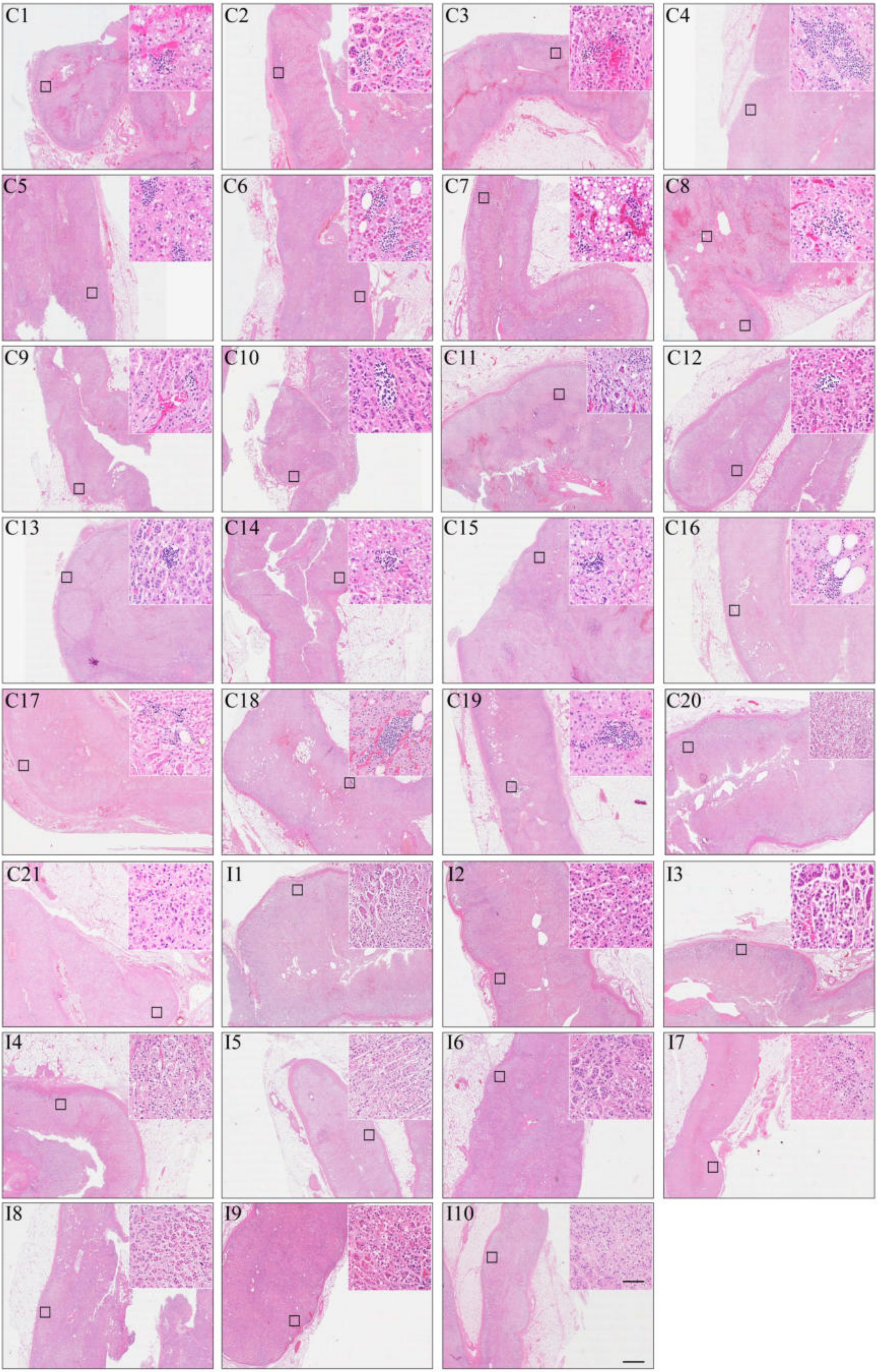

Supplementary Figure 3. H&E images of all adrenal glands (21 COVID-19 patients (C1-21) and 10 influenza patients (I1-10)) are given as an overview (scale bar = 800 μm) and magnified inset (scale bar = 50 μm).

Dilution stage	Volume of virus stock (µl)	MOI
1	20	0.05
2	6.67	0.017
3	2.22	0.0056
4	0.74	0.0019
5	0.25	0.00062
6	0.082	0.00021
7	0.027	6.86E-05
8	0.0091	2.29E-05
9	0.0030	7.62E-06
10	0.0010	2.54E-06
11	0.00034	8.47E-07
12	uninfected (0 µl)	-

Supplementary table 1: Multiplicity of infections (MOIs) for the different dilutions of the inoculum used in Figure 2B:
MOI titration of stock: Cells were plated at a density of 7.5×10^4 cells per well. The highest volume of virus inoculum of 20 µl corresponds to an MOI of 0.05.
