# Supplementary material for: Adrenal tropism of SARS-CoV-2 and adrenal findings in a post-mortem case series of patients with severe fatal COVID-19
Source: Nat Commun. 2022 Mar 24;13:1589. doi: 10.1038/s41467-022-29145-3 (PMC8948269; doi:10.1038/s41467-022-29145-3)
Supplement: Supplementary file 3 — Reporting Summary [file 41467_2022_29145_MOESM3_ESM.pdf]

nature research

Corresponding author(s): Martina Rudelius

Last updated by author(s): Oct 1, 2021

Reporting Summary

Nature Research wishes to improve the reproducibility of the work that we publish. This form provides structure for consistency and transparency in reporting. For further information on Nature Research policies, see our Editorial Policies and the Editorial Policy Checklist.

Statistics

For all statistical analyses, confirm that the following items are present in the figure legend, table legend, main text, or Methods section.

n/a	Confirmed	
☐	☒	The exact sample size (*n*) for each experimental group/condition, given as a discrete number and unit of measurement
☐	☒	A statement on whether measurements were taken from distinct samples or whether the same sample was measured repeatedly
☐	☒	The statistical test(s) used AND whether they are one- or two-sided<br>*Only common tests should be described solely by name; describe more complex techniques in the Methods section.*
☒	☐	A description of all covariates tested
☒	☐	A description of any assumptions or corrections, such as tests of normality and adjustment for multiple comparisons
☐	☒	A full description of the statistical parameters including central tendency (e.g. means) or other basic estimates (e.g. regression coefficient) AND variation (e.g. standard deviation) or associated estimates of uncertainty (e.g. confidence intervals)
☐	☒	For null hypothesis testing, the test statistic (e.g. *F*, *t*, *r*) with confidence intervals, effect sizes, degrees of freedom and *P* value noted<br>*Give P values as exact values whenever suitable.*
☒	☐	For Bayesian analysis, information on the choice of priors and Markov chain Monte Carlo settings
☒	☐	For hierarchical and complex designs, identification of the appropriate level for tests and full reporting of outcomes
☒	☐	Estimates of effect sizes (e.g. Cohen's *d*, Pearson's *r*), indicating how they were calculated

Our web collection on statistics for biologists contains articles on many of the points above.

Software and code

Policy information about availability of computer code

Data collection	InForm 2.4.8
Data analysis	For data analysis SPSS Statistics 27 and MetaboAnalyst 4.0 was used

For manuscripts utilizing custom algorithms or software that are central to the research but not yet described in published literature, software must be made available to editors and reviewers. We strongly encourage code deposition in a community repository (e.g. GitHub). See the Nature Research guidelines for submitting code & software for further information.

Data

Policy information about availability of data

All manuscripts must include a data availability statement. This statement should provide the following information, where applicable:
- Accession codes, unique identifiers, or web links for publicly available datasets
- A list of figures that have associated raw data
- A description of any restrictions on data availability

All data collected for the study and data from the analysis is provided as supplementary files and Source Data files.

Field-specific reporting

Life sciences study design

All studies must disclose on these points even when the disclosure is negative.

Sample size	In this study autopsy samples of patients who died of COVID-19 during the pandemic were analyzed, therefore a sample size calculation could not be performed. At the time point the study was conducted we included all samples that were available and fullfilled our criteria. The sample siize seemed to be sufficient as previous studies conducted on COVID-19 reached statistical significance even with lower sample numbers (e.g. N Engl J Med. 2020, Ackermann et al.; (n=14)). We included specimens of 21 patients who died of COVID-19 with positive PCR-test of SARS-CoV2 from post mortem nasopharyngeal swabs. For comparison archived specimens of 10 (age-matched) patients who died of influenca were choosen. Our statistical tests reached significance.
Data exclusions	No data were excluded.
Replication	For histological and immunohistochemical analysis ten regions of interest were choosen and data was evaluated by two independent experienced pathologists. Exact numbers of samples are given in the figure legends. All attempts at replication were successful.
Randomization	Two experimental groups were compared: patients who died of COVID-19 compared to patients who died of influenza
Blinding	Investigators were blinded to the two different groups.

Reporting for specific materials, systems and methods

We require information from authors about some types of materials, experimental systems and methods used in many studies. Here, indicate whether each material, system or method listed is relevant to your study. If you are not sure if a list item applies to your research, read the appropriate section before selecting a response.

Materials & experimental systems

n/a	Involved in the study	
☐	☒	Antibodies
☐	☒	Eukaryotic cell lines
☒	☐	Palaeontology and archaeology
☒	☐	Animals and other organisms
☐	☒	Human research participants
☒	☐	Clinical data
☒	☐	Dual use research of concern

Methods

n/a	Involved in the study	
☒	☐	ChIP-seq
☒	☐	Flow cytometry
☒	☐	MRI-based neuroimaging

Antibodies

Antibodies used	CYB11B2, Proteintech, rabbit polyclonal, Cat No. 20968-1; CYB5A, ATLAS, rabbit polyclonal, Cat No. HPA058547; Cleaved Caspase 3, Cell signaling technologies, (5A1E), Cat. No. 9664; ACE2, abcam, EPR4435, Cat. No. ab108252; TMPSSR2, Bio SB, BSB-136, Cat No. BSB-3703-05; CD8, Medac, C8/144B, Cat. No. 108M-94; CD4, Medac, EP204, Cat. No. 104R-25; CD34, DAKO, QBEnd, Cat.No. GA63261-2; SF-1, Santa Cruz, A-1, Cat. No. sc-393592; CD68, DAKO, PGM-1, Cat. No. GA61361-2, SARS-Cov-2-s antibody, Genetex, 1A9, GTX632604, SARS-CoV-2- n antibody, cell signalling technologies, Cat. No. 33336, alpha-tubulin, Sigma Aldrich, B-5-1-2, Cat.No. T6074, Il-6, cell signaling technologies, D3K2N, Cat.No.12153
Validation	Before usage for all antibodies an isotype and system control was completed . An adequate negative and positive control was carried along for each staining procedure. Immunohistochemistry for CYB11B2 was tested by the company for human adreanl kidney and liver we used human adrenal tissue (positive) with surroundimg adipose tissue (negative) as control. CYB5A was validated by the company in 44 normal tissues and cancers, we used human adrenal tissue (positive) with surrounding adipose tissue (negative) as control. Cleaved Caspase 3 was validated by CST adapting Uhlen et al. (Nature Methods 2016), as positive and negative control human tonsil was used. ACE2 was validated by abcam with knockout, as positive control human kidney was used. TMPSSR2 was validated by the company with human lung tissue, we used human kidney as positive control. CD8 and CD4 were validated by medac, for further validation we used human tonsil as control. CD34 was validated by different international laboratories according to DAKO , we used tonsil as control tissue. SF-1 was validated by the company with human adrenal tissue, we used human adrenal tissue as control. CD68 was validated by DAKO through collaboration with leading international pathology experts, we used human tonsil as control. SARS-CoV-2 antibody was validated on SARS-CoV-2 infected and non-infected CaCo2 cells that were processed to FFPE blocks. Cell blocks were further used as positive and negative control. Alpha-tubulin was validated by the company for western blot analysis and immunohistochemistry as well as ELISA and immunoprecipitation. Il-6 antibody was validated by the company CST adapting Uhlen et al. (Nature Methods 2016).

Eukaryotic cell lines

Policy information about cell lines

Cell line source(s)	Caco-2 (ATCC; HTB-37); SW13 (ATCC; CCL-105); HAC15 (ATCC; CRL-3301), Vero E6 (ATCC; C1008)

Authentication	Cell lines were not authentical
Mycoplasma contamination	Cell line was tested negative for mycoplasmen
Commonly misidentified lines (See ICLAC register)	No commonly misidentified cell lines were used in this study.

Human research participants

Policy information about studies involving human research participants

Population characteristics	Patient characteristics are given in table 1.
Recruitment	We included patients who died of COVID-19 and were diagnosed of COVID-19 ante mortem. PCR-tests for SARS-CoV-2 were conducted for each patient post mortem. We did not preselect patients in terms of age, gender or comorbidities. The patient cohort consists of patients that were hospitalized due to COVID-19 ante mortem, this might introduce a bias versus critically ill patients.
Ethics oversight	The study was approved by the ethics committe of the LMU (project number 20-1039) and relatives consent was obtained.

Note that full information on the approval of the study protocol must also be provided in the manuscript.
